# Assessment of green technology innovation on energy-environmental efficiency in China under the influence of environmental regulation considering spatial effects

**DOI:** 10.1038/s41598-023-47786-2

**Published:** 2023-11-27

**Authors:** Wei Li, Xiaomin Xu, Shengzhong Huang, Tong Cheng, Mengkai Liu, Can Zhang

**Affiliations:** 1 School of Management, China University of Mining and Technology-Beijing, Beijing, 100083 China; 2 Intelligent Measurement and Control Division, Shandong Giant E-Tech Co., Ltd, Jinan, 250100 China

**Keywords:** Energy conservation, Energy economics, Energy efficiency

## Abstract

Enhancing energy-environmental efficiency (EEE) is crucial for achieving energy conservation and emission reduction goals. Investigating the mechanism through which green technology innovation (GTI) affects EEE and understanding the role of environmental regulation (ER) in this process provides a theoretical basis for efficient utilization of GTI and ER. This study employs a Dynamic Spatial Durbin Model and utilizes panel data from 2003 to 2017 for 30 Chinese provinces to examine the impact of GTI on EEE in the presence of ER. The empirical results reveal: (1) GTI has a U-shaped impact on EEE, primarily driven by SubGI. (2) GTI’s influence on EEE is predominantly reflected in PTE, also stemming from SubGI. (3) The interaction term between ER and GTI is 0.0022, while the GTI coefficient is − 0.0741, and the GTI quadratic term coefficient is 0.0007, all statistically significant. This implies that ER mitigates the negative impact of GTI on EEE while strengthening its positive effect. These findings provide empirical evidence and policy insights for more effectively utilizing GTI and ER to enhance EEE and achieve energy conservation and emissions reduction goals.

## Introduction

Since the initiation of economic reforms and opening-up policies, China’s rapid economic development has led to a growing contradiction between economic growth, energy consumption, and environmental pollution^1^. According to BP Statistical Review of World Energy, in 2019, China’s primary energy consumption reached 141.7 EJ, representing a 4.4% increase compared to the previous year, marking 19 consecutive years of being the fastest-growing energy consumer^2^. High levels of primary energy consumption imply a faster depletion of limited natural energy resources, posing not only a resource depletion issue but also an energy security concern. Furthermore, as an indicator reflecting a nation or region’s sustainable economic development, in 2019, China’s carbon intensity was 48.1% lower than in 2005, but it still exceeded the global average during the period from 1990 to 2019^3^. This underscores the necessity of controlling carbon emission intensity. Therefore, within the context of energy conservation and emissions reduction, exploring ways to enhance EEE becomes a critical measure for reducing energy consumption and elevating carbon intensity, serving as a key driver for achieving sustainable economic development in China.

Green technology innovation (GTI) has garnered significant attention as a crucial means of ecological and environmental protection in various regions. It has shown promising results in controlling industrial energy consumption, reducing energy intensity^4,5^, incentivizing businesses to develop green technologies, lowering the consumption of non-renewable energy sources^6^, and balancing ecological conservation with economic growth^7^. However, a study by Wang and Chen found that the relationship between resource dependency and haze pollution in 263 Chinese prefecture-level cities is complex. While GTI can reduce haze pollution when resource dependency is low or moderate, it may lead to the disappearance of optimization effects when resource dependency is high^8^. Similarly, research by Mongo et al. analyzing environmental innovation data from 15 European countries over 23 years revealed that environmental innovation typically reduces carbon emissions in the long term but may have opposite effects in the short term^9^. This suggests that there may be rebound effects associated with GTI, potentially leading to increased resource consumption and carbon emissions^10^. Therefore, it is crucial to ascertain the impact of GTI on EEE. Furthermore, Zhang et al. pointed out that genuine green technology innovation represents the long-term choices of innovation entities, while strategic green technology innovation reflects their short-term preferences. Environmental regulations were found to exert a greater influence on strategic green innovation^11^. In a study by Xing and Dong, it was revealed that outward foreign direct investment primarily drives reverse green technology innovation in non-invention strategic green innovation categories. Strengthening research and development capabilities and implementing improved environmental regulations can enhance the influence of foreign direct investment on reverse substantive green innovation^12^. This suggests that due to varying innovation motivations, green technology innovation can be categorized into two distinct types: creative substantive green technology innovation and policy-responsive symbolic green innovation. However, current assessments of the impact of GTI on EEE mainly focus on GTI itself, without considering the influence of its components on EEE. In other words, whether the impact of GTI on EEE arises from the development and dissemination of new clean energy technologies or from improvements in technologies related to ecological environmental protection and resource recycling remains unclear.

Furthermore, since 1970, China has formulated and implemented a series of environmental policies^13^ aimed at improving the environment through legal and market mechanisms, such as pollution fees and carbon trading markets^14,15^. The interaction between environmental regulation (ER) and value-added tax motivates enterprises to further reduce the intensity of SO_2_ emissions^16,17^ and promotes innovation in pollution reduction^18^. Therefore, when researching how to enhance Energy-Environmental Efficiency (EEE), considering ER is indispensable. However, current research primarily focuses on the impact of ER on EEE, without considering the role of ER in the mechanism through which GTI affects EEE. In other words, how GTI’s influence on EEE changes due to the implementation of ER is currently unclear.

To address this research gap, this study integrates the definition of GTI, decomposes GTI into Substantial green innovation (SubGI) and Symbolic green innovation (SymGI), and elucidates the underlying mechanisms through which GTI affects EEE. Specifically, it clarifies whether the impact of GTI on EEE is driven by the development and promotion of new clean energy technologies or by improvements in ecological environmental protection and resource recycling technologies. Secondly, it introduces the interaction effects of ER with GTI, SubGI, and SymGI, elucidating the role of ER in the mechanisms through which GTI, SubGI, and SymGI influence EEE. Lastly, it incorporates spatial factors by employing spatial econometric models to explore the impact of GTI on EEE under the influence of ER, resulting in more realistic and reliable outcomes.

This study makes several potential contributions to the existing literature. Firstly, by decomposing GTI, the study delves deeper into the mechanisms of GTI, enriching the research on the impact of GTI on EEE and shedding light on the black box of how GTI operates. Secondly, by simultaneously considering the moderating role of ER and spatial spillover effects, the study aligns with the real-world context, providing a theoretical basis and reference for China and other developing countries to more accurately utilize GTI and ER for energy conservation and emissions reduction.

The remaining sections of this paper are organized as follows: Section "Literature review" provides a review of relevant literature. Section "Data and methodology" describes the data, models, and methods used in the study. Section "Results and discussion" presents the analysis and results. Finally, Section "Conclusion and policy implications" summarizes the research findings and offers policy recommendations.

## Literature review

Technology shapes the future of energy, and technological innovation creates the future of energy. Under the constraints of China’s dual carbon targets, achieving energy conservation, emission reduction, and improving EEE has become an urgent priority. In the analysis of energy and the environment, EEE is considered the most promising tool for establishing a harmonious relationship between economic growth and resource consumption^19^. Therefore, the driving mechanisms of EEE have garnered widespread attention. The structure of the literature review is outlined in Table 1.

**Table 1:** Literature review structure.

Indicators	Dimension	Summary	References
GTI	GTI has a positive impact	Improved environmental performance, profitability, core competitiveness of enterprises, total factor carbon productivity in high-income economies and carbon performance	^20–24^
	Non-linear variation in the impact of GTI	A critical point is reached prior to a boost in green productivity and green economic efficiency, with an inverted U-shaped effect on regional carbon emissions	^28–30^
ER	ER has a positive effect	Promoting green transformation of enterprises, fostering long-term economic growth, increasing green innovation outputs, improving energy efficiency in the environment and green technological efficiency	^11,14,33–36^
	ER has a moderating effect	Enhancing the impact of green knowledge innovation on CO_2_ emissions	^11,26^
EEE	EEE has a positive effect	Reducing pollution emissions, enhancing the sustainability of resource utilization and stimulating technological innovation to effectively counter environmental problems and promote sustainable development	^38–41^
	EEE is affected by multiple factors	Influenced by technological innovation, policies and regulations, FDI absorptive capacity, level of economic development, industrial and energy structure, etc	^42–46^
	EEE consists of SE, PTE	EEE is the multiplication of SE and PTE	^47–49^

GTI, as a major driver of sustainable development, has garnered widespread attention from scholars across various fields. Firstly, green innovation not only enhances a company’s environmental performance^20^, profitability^21^, and core competitiveness of enterprises^22^, but also contributes to total factor carbon productivity in high-income economies^23^, and carbon performance^24^. The main reason behind this is that green innovation propels companies to adopt more environmentally friendly production and operational methods, reducing resource waste and pollution emissions, thus improving environmental performance. Although GTI brings various beneficial impacts, the specific part of this impact generated by GTI may vary depending on the industry or context^25^. For instance, ER increase the effect of green knowledge innovation on CO_2_ reduction^26^, further encouraging heavy-polluting enterprises to preferentially focus on substantial green technology innovation during their green transformation^27^. However, the fundamental reasons behind the generation of these impacts by GTI have not been thoroughly explored. Secondly, there are also some contrasting research findings suggesting that technological innovation only exerts a driving effect on green productivity^28^ and green economic efficiency^29^ after reaching critical points, displaying non-linear changes. Similarly, the impact of GTI on regional carbon emissions^30^ exhibits an inverted U-shaped change of initially increasing and then decreasing. Hence, the impact of GTI is not constant, and this phenomenon may be related to the intrinsic drivers of GTI, wherein different intrinsic factors may be at work during different periods. Nevertheless, some similar studies overlook these intrinsic driving factors of GTI.^31,32^.

ER, as a political tool, can drive corporate green transformation^11^, promote long-term economic growth^14^, enhance green innovation output^33^, improve EEE^34,35^, and green technological efficiency^36^. The main rationale behind this is that moderate ER can partially or fully offset enterprise innovation costs through the compensating effects of green innovation, thus increasing enterprise innovation output. However, the implementation of ER can also have an impact on other factors^11^. For example, ER can significantly increase the impact of green knowledge innovation on CO_2_ emissions, but the impact on green process innovation is not as pronounced^26^. Therefore, ER is a crucial consideration in exploring the mechanism of impact. Additionally, environmental policies and carbon emissions also have spatial spillover effects^37^, and studying spatial factors is more realistic.

EEE is a vital concept in the fields of green economy and sustainable development. On one hand, improving EEE can reduce pollution emissions^38^, enhance sustainable resource utilization^39^, stimulate of technological innovation, effectively address environmental issues^40^ and promoting sustainable development^41^. On the other hand, EEE is influenced by various factors such as technological innovation^42^, policy regulations^43^, absorptive capacity of foreign direct investment^44^, economic development level^45^, industrial structure^45^, and energy structure^46^. EEE is a complex indicator that requires in-depth research and comprehensive consideration. Furthermore, EEE consists of scale efficiency (SE) and pure technical efficiency (PTE). In simple terms, EEE is the product of these two efficiencies^47^. SE is the production efficiency influenced by scale factors^48^, while PTE is the production efficiency affected by management and technological factors^49^. Therefore, it is essential to explore which part of EEE is affected by various influencing factors.

In summary, despite extensive research in the field of energy-environment management on EEE, GTI and ER, there exists a research gap regarding the underlying mechanisms of GTI and the moderating role of ER in this context. China’s energy structure necessitates higher energy utilization efficiency to achieve macro-level energy-saving goals, making it crucial to consider how to enhance energy utilization efficiency through GTI, which serves as a cornerstone in achieving dual carbon objectives. Addressing the limitations in evaluating GTI’s impact on EEE, this study decomposes GTI into SubGI and SymGI, introduces the interaction terms of ER with GTI, SubGI, and SymGI to explore the sources of GTI’s impact on EEE and the moderating effects of ER on this impact and its sources. Empirical analysis is conducted using spatial Durbin models, incorporating spatial spillover effects, which better align with real-world development scenarios.

## Data and methodology

Given that the 16th National Congress of the Communist Party of China was held in November 2002, it was emphasized that “development is an unyielding principle, and every opportunity must be seized to accelerate progress”. This prompted provincial governments to vigorously promote regional industrialization and urbanization. However, the accelerated process of industrialization and urbanization has led to a significant increase in energy demand and consumption, exacerbating the conflict between energy and the environment. Therefore, this study takes the year 2003 as its starting point and first calculates the EEE of each province in China. Subsequently, it investigates the impact of GTI on EEE under the influence of ER. However, the calculation of EEE requires the input indicator of capital stock, which is measured by gross fixed capital formation in this research. Unfortunately, this data is not available in the China Statistical Yearbook after 2017. To ensure data availability, the study ultimately selects data from 30 provinces and regions in China (excluding Tibet, Hong Kong, Macao, and Taiwan) from 2003 to 2017 for analysis.

**Figure 1:**
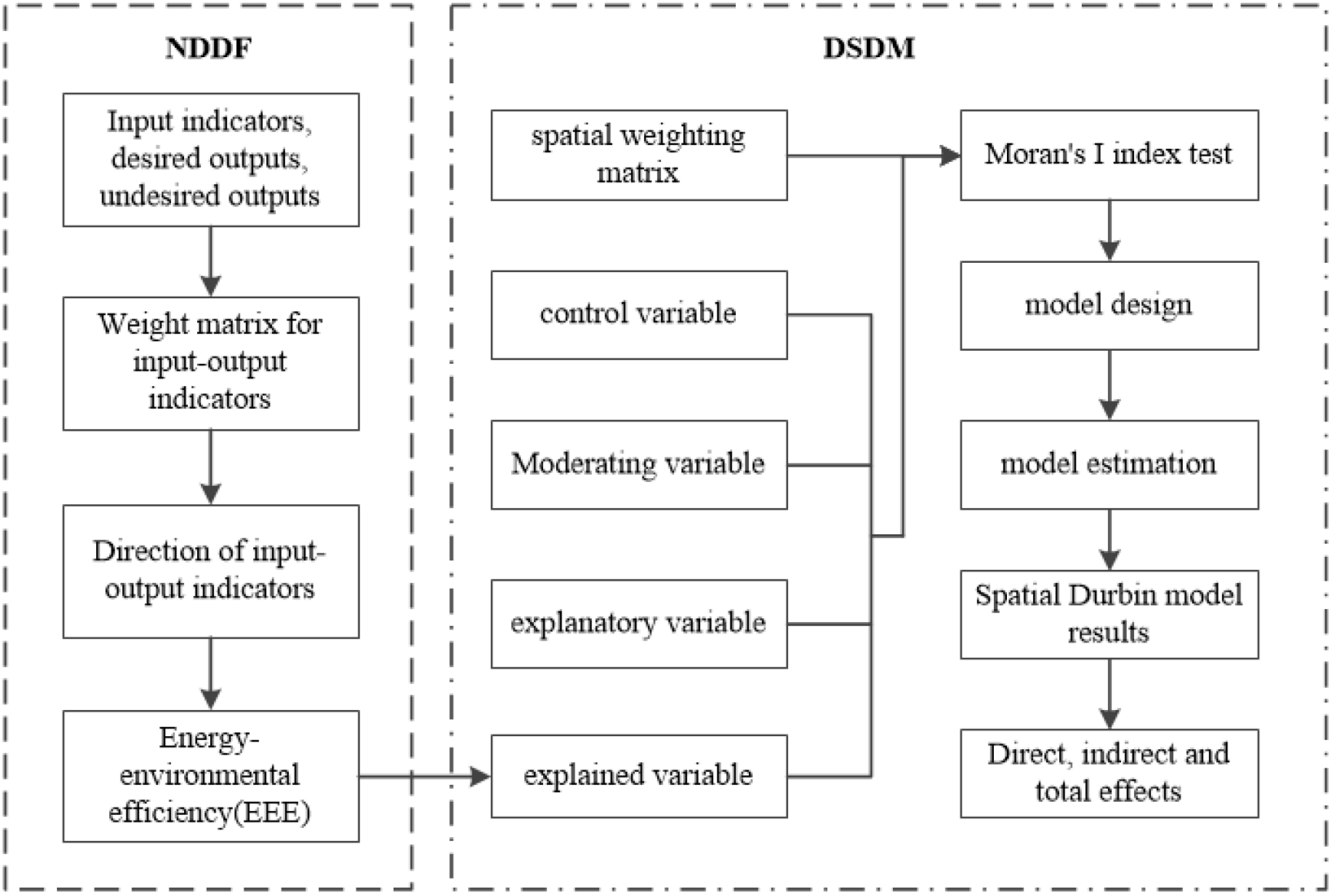
Flowchart of the method.

A two-step approach was used to investigate the impact of GTI on EEE under the influence of ER from 2003 to 2017. Firstly, EEE in 30 provinces was measured. Secondly, the impact of GTI on EEE under the influence of ER was analyzed. It is shown in Fig. 1. This article considers EEE, SE, and PTE as explanatory variables, GTI, SubGI, and SymGI as explanatory variables, and ER as a moderating variable. Besides, gross domestic product per capita (PGDP), industrial structure (IS), foreign direct investment (FDI), energy consumption structure (ECStruc), urbanization level (Urban), R&D investment intensity (RDI), energy intensity (EI), fixed asset investment (Fix), R&D personnel input (RDP) are used as control variables.

### Data description

Green technology innovation (GTI). GTI refers to the development and application of new products and technologies aimed at environmental protection, pollution reduction, energy and resource conservation, and promoting sustainable development^1,50^. On the other hand, green patents are inventions, utility models, and design patents with the subject of invention related to resource conservation, energy efficiency, and pollution prevention. Considering the alignment with the definition of GTI, design patents only cover product shapes and patterns, hence this study adopts the total number of green invention patents and green utility model patents to measure GTI^51^. As the patent granting process in China is known to be time-consuming, using the total number of patent applications can promptly and accurately reflect the willingness and motivation of enterprises to engage in GTI. Additionally, based on the essence of GTI definition, which includes both the development and promotion of new clean energy technologies, as well as improvements in ecological environment protection and resource recycling technologies, this research attempts to decompose GTI into SubGI and SymGI.

Substantive green innovation (SubGI). Substantive green technology innovation is focused on the development and adoption of environmentally beneficial technologies that result in significant reductions in resource consumption and carbon emissions. Green invention patents exhibit creativity, novelty, and energy-efficient features, aligning with the fundamental principles of substantive green innovation^52^. As a result, this study utilizes the quantity of green invention patent applications as a metric to assess SubGI^11,53^.

Symbolic green innovation (SymGI). Symbolic green innovation is aimed at responding to government environmental policies, emphasizing improvements to existing technologies but typically lacking significant positive environmental impacts. Green utility model patents refer to product or process improvements that feature energy-saving and emission reduction characteristics, aligning with the core principles of symbolic green technology innovation^52,54^. Hence, this study employs the quantity of green utility model patent applications as a metric to assess SymGI^11,53^.

Environmental regulation (ER). Based on the polluter-pays principle, China began to levy emission charges in 1982^55^ By imposing charges on enterprises and individuals that emit pollutants, it can incentivize them to adopt more environmentally-friendly measures, reduce emissions of pollutants, and consequently mitigate environmental pollution and resource wastage. In light of this, the emission charges to GDP is used as a measure of ER.

This study integrates the exercises of several scholars to take the PGDP^56^, IS^12^, FDI^44^, ECStruc^52^, Urban^56^, RDI^57^, EI^58^, Fix^59^, RDP as control variable, the definition of each variables is shown in Table 10.

### Non-radial direction distance function

In this section, the study concentrates on the computation of the dependent variable, EEE. Due to the flexibility of DEA in not requiring a specific functional form, it can effectively measure the EEE of DMU^60,61^. In this context, DMU refers to the 30 provinces from 2003 to 2017.

Non-radial Directional Distance Function (NDDF) is a variant of the DEA method that offers several advantages in assessing EEE. Firstly, it allows for the simultaneous consideration of multiple input and output indicators, enabling a more comprehensive evaluation of regional efficiency. Secondly, it introduces directionality, which determines the optimization direction, leading to more precise assessment results. Additionally, it permits the assignment of different weights to different input and output indicators when calculating the distance function, reflecting their importance in the evaluation process. In summary, NDDF demonstrates broader applicability when assessing EEE as it comprehensively accounts for multiple indicators and their respective weights, contributing to a more accurate assessment of the efficiency levels of businesses or regions.

The NDDF function is defined based on the principle of output expansion while minimizing pollutant emissions as follows^62^:

1 $$\overrightarrow{D}\left(K,L,E,Y,C;g\right)=sup\left\{{w}^{T}\beta :\left(\left(K,L,E,Y,C\right)+g\cdot diag\left(\beta \right)\right)\in P\right\}$$

where K, L, E are input variables, Y is desired output and C is undesired output. The input and output variables are shown as follows:

Input indicator: Capital (K). Estimate the capital stock using the perpetual inventory method^34^. Labor (L). Measures labor through the number of people employed at the end of each year in each region. Energy (E). Measured using the consumption of tons of standard coal in each region.

Expected output: Total output value of each province (Y). Converted from nominal GDP to 2003 constant price GDP through a price index.

Unintended output: CO_2_ emissions (C). CO_2_ emissions are calculated from the calorific value of consumption of nine energy sources: raw coal, coking coal, crude oil, gasoline, kerosene, diesel fuel, fuel oil, natural gas, and electricity.

Additionally, ${w}^{T}=\left({w}_{K},{w}_{L},{w}_{E},{w}_{Y},{w}_{C}\right)$ denotes the vector of weights for each input–output variable; ${\beta }^{T}=\left({\beta }_{K},{\beta }_{L},{\beta }_{E},{\beta }_{Y},{\beta }_{C}\right)$ denotes the slack vector of the proportion in which each input–output variable can be expanded or contracted; ${g}^{T}=\left({g}_{K},{g}_{L},{g}_{E},{g}_{Y},{g}_{C}\right)$ denotes the direction vector of the direction of input and output changes (i.e., expansion or contraction), and $diag\left(\beta \right)$ denotes the diagonalization of the $\beta$ vector.

EEE examines the maximum shrinkage ratio of energy inputs, undesired outputs, and the maximum expansion ratio of desired outputs with constant capital and labor inputs. Therefore, the weight vector is set to ${w}^{T}=\left(\mathrm{0,0},\frac{1}{3},\frac{1}{3},\frac{1}{3}\right)$ , and the respective direction vector is $g=\left(\mathrm{0,0},-\mathrm{E},\mathrm{Y},-\mathrm{C}\right)$ , and the corresponding linear programming problem is as follows:

2 $$\overrightarrow{D}\left(K,L,E,Y,C\right)=max\left\{\frac{1}{3}{\beta }_{E}+\frac{1}{3}{\beta }_{Y}+\frac{1}{3}{\beta }_{C}\right\}$$

Similarly, the optimal solution for the relaxation variable ${\beta }_{it}^{*}={\left({\beta }_{it,K}^{*},{\beta }_{it,L}^{*},{\beta }_{it,E}^{*},{\beta }_{it,Y}^{*},{\beta }_{it,C}^{*}\right)}^{T}$ and thus the EEE for each province and region is:

3 $${EEE}_{it}=\frac{1}{6}\left(\frac{{Y}_{it}/{E}_{it}}{\left({Y}_{it}+{\beta }_{it,Y}^{*}{Y}_{it}\right)/\left({E}_{it}+{\beta }_{it,E}^{*}{E}_{it}\right)}+\frac{{Y}_{it}/{C}_{it}}{\left({Y}_{it}+{\beta }_{it,Y}^{*}{Y}_{it}\right)/\left({C}_{it}+{\beta }_{it,Y}^{*}{C}_{it}\right)}\right)$$

### Dynamic spatial Durbin model

The literature review mentions that ER has spatial spillover effects and that CO_2_ generates spatial spillover due to geographic boundaries or natural winds, among other issues. In order to further illustrate the existence of spatial effects among the research variables, this article firstly analyzes the explanatory and interpreted variables by using Moran’s I index. As in Eq. (4):

4 $${\mathrm{Moran}}^{\mathrm{^{\prime}}}\mathrm{s I}=\frac{\mathrm{n}\sum_{\mathrm{i}=1}^{30}\sum_{\mathrm{j}=1}^{30}{\mathrm{W}}_{\mathrm{ij}}\left({\mathrm{x}}_{\mathrm{it}}-{\overline{\mathrm{x}}}_{\mathrm{t}}\right)\left({\mathrm{x}}_{\mathrm{jt}}-{\overline{\mathrm{x}}}_{\mathrm{t}}\right)}{\sum_{\mathrm{i}=1}^{30}{\left({\mathrm{x}}_{\mathrm{it}}-{\overline{\mathrm{x}}}_{\mathrm{t}}\right)}^{2}\sum_{\mathrm{i}=1}^{30}\sum_{\mathrm{j}=1}^{30}{\mathrm{W}}_{\mathrm{ij}}}$$

where ${\overline{x}}_{t}=\frac{1}{30}\sum_{i=1}^{30}{x}_{it}$, and ${x}_{it}$ are the variable to be spatially autocorrelated tested. ${W}_{ij}$ are the spatial weight matrix. To improve the accuracy of the results, three spatial weight matrices are considered in this study: the geographic neighborhood weight matrix (${W}_{a}$), the geographic distance weight matrix (${W}_{b}$), and the economic geographic distance weight matrix (${W}_{c}$).

Next, except for the spatial effect, there is also a time lag in CO_2_ emissions^63,64^. Thus, in order to make the empirical results more reliable, the dynamic spatial Durbin model (DSDM), which takes time and space into consideration, is adopted as the empirical model in this paper. As shown in Eqs. (5) to (10). Where, in order to have a more in-depth understanding of the effect of GTI on EEE, this article adds the quadratic term of GTI ((${\mathrm{GTI}}_{it}$)^2^) into the model, the $\sum_{j=1}^{30}{W}_{ij}{lnEEE}_{it}$, $\sum_{j=1}^{30}{W}_{ij}{lnGTI}_{it}$, $\sum_{j=1}^{30}{W}_{ij}{lnER}_{it}$, $\sum_{j=1}^{30}{W}_{ij}{lnSubGI}_{it}$, $\sum_{j=1}^{30}{W}_{ij}{lnSymGI}_{it}$, $\sum_{j=1}^{30}{W}_{ij}{lnPTE}_{it}$ and $\sum_{j=1}^{30}{W}_{ij}{lnSE}_{it}$ are spatial variables, ${lnControls}_{it}$ is a control variables.

5 $${lnEEE}_{it}={\alpha }_{0}+{\lambda }_{0}{lnEEE}_{it-1}+{\rho }_{0}\sum_{j=1}^{30}{W}_{ij}{lnEEE}_{jt}+{\iota }_{0}\sum_{j=1}^{30}{W}_{ij}{lnGTI}_{jt}+{\varpi }_{1}{lnGTI}_{it}+{\varpi }_{2}{\left({lnGTI}_{it}\right)}^{2}+\varpi {lnControls}_{it}+{\mu }_{i}+{\varepsilon }_{it}$$

6 $${lnEEE}_{it}={\alpha }_{0}+{\lambda }_{1}{lnEEE}_{it-1}+{\rho }_{1}\sum_{j=1}^{30}{W}_{ij}{lnEEE}_{jt}+{\iota }_{1}\sum_{j=1}^{30}{W}_{ij}{lnGTI}_{jt}+{\alpha }_{1}{lnGTI}_{it}+{\alpha }_{2}{\left({lnGTI}_{it}\right)}^{2}+{\vartheta }_{1}\sum_{j=1}^{30}{W}_{ij}{lnER}_{jt}{+\alpha }_{3}{lnER}_{it}+{\kappa }_{1}\sum_{j=1}^{30}{W}_{ij}{\mathrm{ln}\left(GTIER\right)}_{jt}+{\alpha }_{4}{\mathrm{ln}\left(GTIER\right)}_{it}+\alpha {lnControls}_{it}+{\mu }_{i}+{\varepsilon }_{it}$$

7 $${lnEEE}_{it}={\alpha }_{0}+{\lambda }_{2}{lnEEE}_{it-1}+{\rho }_{2}\sum_{j=1}^{30}{W}_{ij}{lnEEE}_{jt}+{\nu }_{1}\sum_{j=1}^{30}{W}_{ij}{lnSubGI}_{jt}+{\beta }_{1}{lnSubGI}_{it}+{\pi }_{1}\sum_{j=1}^{30}{W}_{ij}{lnSymGI}_{jt}+{\beta }_{2}{lnSymGI}_{it}+{\vartheta }_{2}\sum_{j=1}^{30}{W}_{ij}{lnER}_{jt}{+\beta }_{3}{lnER}_{it}+{\xi }_{1}\sum_{j=1}^{30}{W}_{ij}{\mathrm{ln}\left(SubGIER\right)}_{jt}+{\beta }_{4}{\mathrm{ln}\left(SubGIER\right)}_{it}+{\zeta }_{1}\sum_{j=1}^{30}{W}_{ij}{\mathrm{ln}\left(SymGIER\right)}_{jt}+{\beta }_{5}{\mathrm{ln}\left(SymGIER\right)}_{it}+\beta {lnControls}_{it}+{\mu }_{i}+{\varepsilon }_{it}$$

8 $${lnPTE}_{it}={\alpha }_{0}+{\lambda }_{3}{lnPTE}_{it-1}+{\varphi }_{1}\sum_{j=1}^{30}{W}_{ij}{lnPTE}_{jt}+{\iota }_{2}\sum_{j=1}^{30}{W}_{ij}{lnGTI}_{jt}+{\gamma }_{1}{lnGTI}_{it}+{\gamma }_{2}{\left({lnGTI}_{it}\right)}^{2}+{\vartheta }_{3}\sum_{j=1}^{30}{W}_{ij}{lnER}_{jt}{+\gamma }_{3}{lnER}_{it}+{\kappa }_{2}\sum_{j=1}^{30}{W}_{ij}{\mathrm{ln}\left(GTIER\right)}_{jt}+{\gamma }_{4}{\mathrm{ln}\left(GTIER\right)}_{it}+\gamma {lnControls}_{it}+{\mu }_{i}+{\varepsilon }_{it}$$

9 $${lnPTE}_{it}={\alpha }_{0}+{\lambda }_{4}{lnPTE}_{it-1}+{\varphi }_{2}\sum_{j=1}^{30}{W}_{ij}{lnPTE}_{jt}+{\nu }_{2}\sum_{j=1}^{30}{W}_{ij}{lnSubGI}_{jt}+{\delta }_{1}{lnSubGI}_{it}+{\pi }_{1}\sum_{j=1}^{30}{W}_{ij}{lnSymGI}_{jt}+{\delta }_{2}{lnSymGI}_{it}+{\vartheta }_{4}\sum_{j=1}^{30}{W}_{ij}{lnER}_{jt}{+\delta }_{3}{lnER}_{it}+{\xi }_{2}\sum_{j=1}^{30}{W}_{ij}{\mathrm{ln}\left(SubGIER\right)}_{jt}+{\delta }_{4}{\mathrm{ln}\left(SubGIER\right)}_{it}+{\zeta }_{2}\sum_{j=1}^{30}{W}_{ij}{\mathrm{ln}\left(SymGIER\right)}_{jt}+{\delta }_{5}{\mathrm{ln}\left(SymGIER\right)}_{it}+\delta {lnControls}_{it}+{\mu }_{i}+{\varepsilon }_{it}$$

10 $${lnSE}_{it}={\alpha }_{0}+{\lambda }_{5}{lnSE}_{it-1}+{\phi }_{1}\sum_{j=1}^{30}{W}_{ij}{lnSE}_{jt}+{\iota }_{3}\sum_{j=1}^{30}{W}_{ij}{lnGTI}_{jt}+{\eta }_{1}{lnGTI}_{it}+{\eta }_{2}{\left({lnGTI}_{it}\right)}^{2}+{\vartheta }_{5}\sum_{j=1}^{30}{W}_{ij}{lnER}_{jt}{+\eta }_{3}{lnER}_{it}+{\kappa }_{3}\sum_{j=1}^{30}{W}_{ij}{\mathrm{ln}\left(GTIER\right)}_{jt}+{\eta }_{4}{\mathrm{ln}\left(GTIER\right)}_{it}+\eta {lnControls}_{it}+{\mu }_{i}+{\varepsilon }_{it}$$

**Figure 2:**
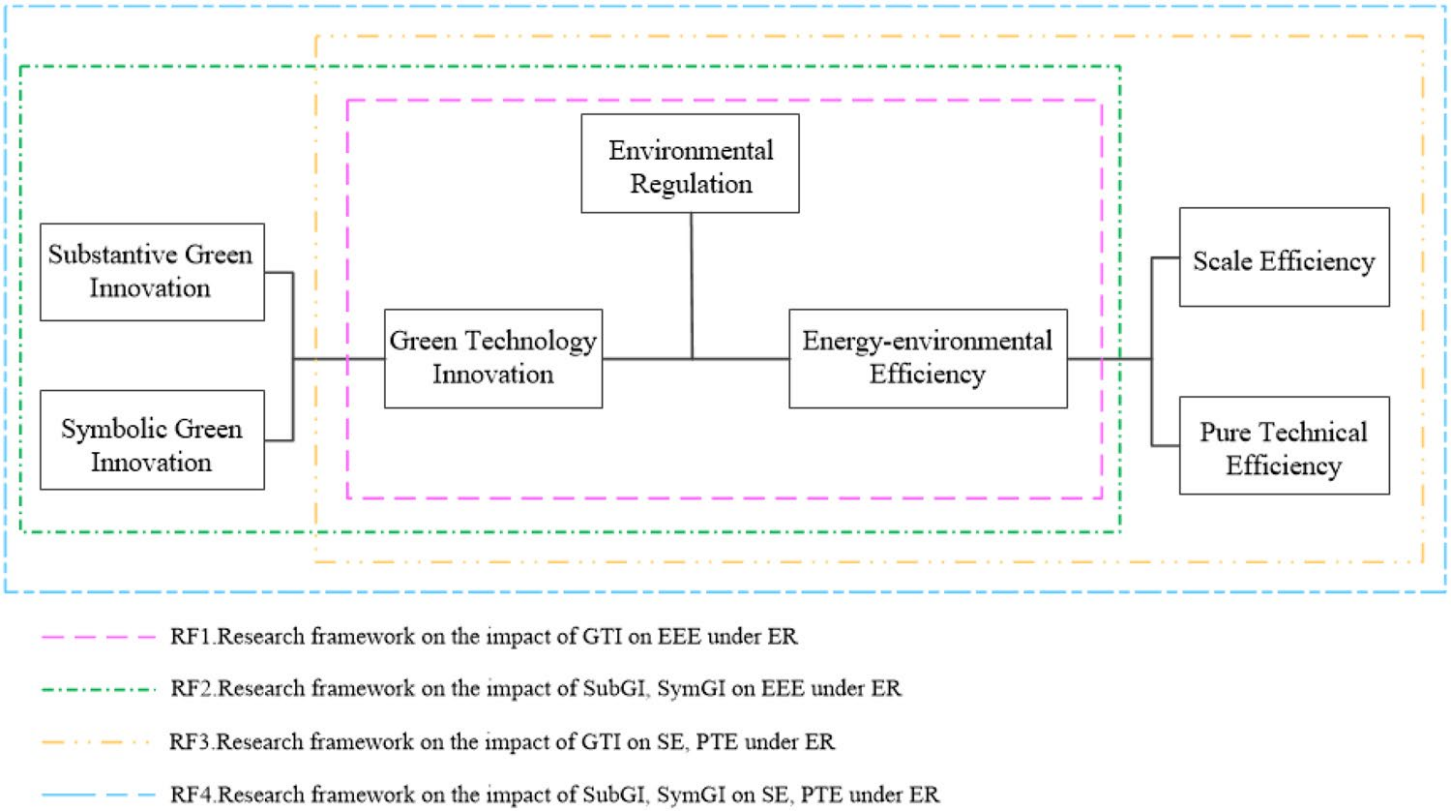
research framework.

Model (5) is the model of the impact of GTI on EEE without considering ER. Model (6) is the impact of GTI on EEE under the influence of ER, corresponding to RF1 in Fig. 2 of the research framework. Model (7) is the impact of SubGI and SymGI on EEE under the influence of ER, corresponding to RF2 in Fig. 2 of the research framework. Model (8) and model (10) are the effects of GTI on PTE and SE under the influence of ER, corresponding to RF3 in Fig. 2 of the research framework. Model (9) is the effects of SubGI and SymGI on PTE under the influence of ER, corresponding to RF4 in Fig. 2 of the research framework. Where ${\alpha }_{0}$ denote the constant term; ${\lambda }_{i}$ is the time-lag effects of the explained variables; ${\varpi }_{i}$, ${\alpha }_{i}$, ${\beta }_{i}$, ${\gamma }_{i}$, ${\delta }_{i}$, and ${\eta }_{i}$ are the coefficients of the independent and control variables; ${\mu }_{i}$ is the individual fixed effect; ${\varepsilon }_{it}$ refers to the error term. $\uprho$, $\mathrm{\varphi }$, $\upphi$, $\upiota$, $\upnu$, $\uppi$, $\mathrm{\vartheta }$, $\upkappa$, represent the coefficients of spatial spillover effects of the explained variables, explanatory variables and interaction terms, respectively.

Due to the panel nature of the data, involving both time series and cross-sectional dimensions, using Ordinary Least Squares (OLS) for estimation would result in biased and inconsistent estimates^19^. Furthermore, Generalized Least Squares (GLS) can be applied to panel data but does not account for spatial effects, leading to biased estimates as well. Finally, spatial autoregressive model (SAR), spatial error model (SEM) and spatial Durbin’s model (SDM) all consider the influence of spatial factors. However, SAR neglects cross-variable autocorrelation, while SEM overlooks spatial dependency among variables. Hence, this study opted for SDM as the base model, extending it to incorporate lag effects, thereby forming a DSDM.

## Results and discussion

### The spatial autocorrelation analysis

The spatial correlations for EEE, PTE, SE, ER, GTI, SubGI, and SymGI were calculated under three spatial weight matrices, and the results are presented in Table 2.

**Table 2:** The global Moran’s I values of variables from 2003 to 2017.

	EEE			PTE			SE			ER	
	${W}_{a}$	${W}_{b}$	${W}_{c}$	${W}_{a}$	${W}_{b}$	${W}_{c}$	${W}_{a}$	${W}_{b}$	${W}_{c}$	${W}_{a}$	${W}_{b}$
2003	0.341***	0.107***	0.270***	0.012	− 0.013	0.067	0.012	− 0.013	0.067	0.121*	0.121
2004	0.376***	0.118***	0.288***	0.038	− 0.020	0.194***	0.038	− 0.020	0.194***	0.165*	0.165
2005	0.392***	0.121***	0.237***	0.083	− 0.010	0.206***	0.083	− 0.010	0.206***	0.145*	0.145
2006	0.422***	0.127***	0.225***	0.182**	0.013*	0.155**	0.182**	0.013*	0.155**	0.149*	0.149
2007	0.412***	0.120***	0.203***	0.215**	0.021*	0.171**	0.215**	0.021*	0.171**	0.131*	0.131*
2008	0.408***	0.117***	0.198***	0.170**	0.012*	0.240***	0.170**	0.012*	0.240***	0.174**	0.174**
2009	0.391***	0.109***	0.198***	0.252**	0.032**	0.122**	0.252**	0.032**	0.122**	0.205**	0.205**
2010	0.383***	0.102***	0.197***	0.235**	0.028**	0.178***	0.235**	0.028**	0.178***	0.210**	0.210**
2011	0.377***	0.097***	0.209***	0.233**	0.026**	0.158**	0.233**	0.026**	0.158**	0.192**	0.192**
2012	0.398***	0.101***	0.219***	0.236**	0.028**	0.167**	0.236**	0.028**	0.167**	0.198**	0.198**
2013	0.399***	0.099***	0.240***	0.214**	0.023**	0.163**	0.214**	0.023**	0.163**	0.177**	0.177**
2014	0.400***	0.102***	0.240***	0.214**	0.026**	0.166**	0.214**	0.026**	0.166**	0.166**	0.166**
2015	0.373***	0.097***	0.244***	0.193**	0.022**	0.154**	0.193**	0.022**	0.154**	0.233**	0.233***
2016	0.395***	0.104***	0.247***	0.234**	0.034**	0.173***	0.234**	0.034**	0.173***	0.244***	0.244***
2017	0.412***	0.110***	0.259***	0.260***	0.043**	0.178***	0.260***	0.043**	0.178***	0.273***	0.273***

	ER	GTI			SubGI			SymGI			
	${W}_{c}$	${W}_{a}$	${W}_{b}$	${W}_{c}$	${W}_{a}$	${W}_{b}$	${W}_{c}$	${W}_{a}$	${W}_{b}$	${W}_{c}$	
2003	0.263***	0.150*	0.036**	0.227***	0.140*	0.029**	0.163***	0.136*	0.023**	0.261***	
2004	0.207***	0.157*	0.025**	0.215***	0.139*	0.020**	0.136**	0.154*	0.019*	0.266***	
2005	0.156**	0.176**	0.033**	0.195***	0.169**	0.030**	0.118**	0.170**	0.026**	0.254***	
2006	0.128**	0.170**	0.033**	0.207***	0.169**	0.028**	0.136**	0.146*	0.026**	0.269***	
2007	0.005	0.194**	0.037**	0.199***	0.180**	0.026**	0.128**	0.173**	0.032**	0.258***	
2008	0.003	0.202**	0.033**	0.214***	0.139*	0.008*	0.132**	0.235**	0.047***	0.285***	
2009	0.053	0.236**	0.041**	0.233***	0.177**	0.015*	0.155**	0.263***	0.057***	0.292***	
2010	0.074	0.254***	0.042**	0.239***	0.171**	0.008*	0.154**	0.307***	0.064***	0.302***	
2011	0.049	0.277***	0.049***	0.230***	0.191**	0.015*	0.151**	0.327***	0.071***	0.288***	
2012	0.087*	0.277***	0.052***	0.232***	0.200**	0.018**	0.167***	0.322***	0.074***	0.273***	
2013	0.054	0.231**	0.039**	0.215***	0.187**	0.017*	0.164***	0.261***	0.058***	0.257***	
2014	0.105*	0.242***	0.041**	0.213***	0.196**	0.021**	0.166***	0.272***	0.057***	0.254***	
2015	0.141**	0.291***	0.058***	0.236***	0.238***	0.037**	0.193***	0.307***	0.067***	0.258***	
2016	0.136**	0.292***	0.060***	0.256***	0.256***	0.051***	0.233***	0.300***	0.058***	0.264***	
2017	0.098*	0.225**	0.036**	0.250***	0.197**	0.051***	0.236**	0.225**	0.037**	0.248***	

***, **, * indicate significance at the level of 1%, 5%, 10%.

Table 2 reveals that from 2003 to 2017, the Moran’s I values for EEE, GTI, SubGI, and SymGI were all significantly positive, indicating spatial clustering and substantial positive spatial correlation among provinces. Additionally, it was observed that, except for the years 2003, 2004, and 2005 under conditions ${W}_{a}$ and ${W}_{b}$, where Moran’s I values for PTE and SE were positive but not significant, all other periods exhibited significantly positive Moran’s I values, signifying a strong positive spatial correlation between PTE and SE. Finally, the Moran’s I results for ER demonstrated that under condition ${W}_{a}$ all Moran’s I values were significantly positive, suggesting a robust positive spatial correlation among ER. Under condition ${W}_{b}$, apart from the years 2003 to 2006 with positive but insignificant Moran’s I values, all other results were significantly positive. Under condition ${W}_{c}$, Moran’s I values for the years 2007 to 2012 were positive but insignificant, while all other results were significantly positive. In summary, the majority of Moran’s I values for ER were significantly positive, indicating a pronounced spatial dependence among ER. Overall, there is substantial spatial correlation between explanatory and dependent variables, underscoring the necessity to account for spatial effects in the study, which aligns with real-world dynamics.

### Estimated results of the effect of GTI on EEE under the impact of ER

This section employs the DSDM to estimate the impact of GTI on EEE without considering the influence of ER (model 5) and the impact of GTI on EEE under the influence of ER (model 6), as presented in Table 3. Results from Table 3 indicate that under all three spatial weight matrices, the coefficients for the variables are generally significant. Under the ${\mathrm{W}}_{b}$ weight matrix, the spatial correlation coefficients and sigma coefficients are also significant, suggesting the suitability of the DSDM model for this estimation. The lagged term coefficient of EEE ($\mathrm{L}.\mathrm{lnEEE}$) and the spatial spillover term coefficient of EEE under weight ${\mathrm{W}}_{b}$ ($\mathrm{L}.\mathrm{WlnEEE}$) are both significantly positive, indicating a clear pattern of continuity, accumulation, and high interdependence among EEE in various provinces of China. In addition, under all three weight matrices, the coefficient for the quadratic term of GTI is significantly positive, indicating a U-shaped relationship between GTI and EEE. One possible explanation for this pattern is that the transformation of GTI takes time and requires significant initial investments in research and improvement, leading to relatively stable energy consumption and CO_2_ emissions, resulting in a decline in EEE. However, once the GTI are widely adopted, energy consumption and CO_2_ emissions are substantially reduced, leading to improved EEE. Furthermore, the study finds that the direct effect coefficients of ER on EEE and the spatial spillover effect coefficients are opposite in sign. The direct interaction effect coefficients between ER and GTI, as well as the spatial interaction effect coefficients between ER and GTI, are also opposite in sign. This makes it challenging to directly assess the impact of ER on EEE and the role of ER in this impact. Therefore, the study decomposed the model results. Since short-term estimates are particularly valuable for informing national energy controls and energy policies, this study focuses on analyzing short-term effect results, as shown in Tables 4 and 5.

**Table 3:** Results of the impact of GTI on EEE.

Model	(5)			(6)		
Weight	${\mathrm{W}}_{a}$	${\mathrm{W}}_{b}$	${\mathrm{W}}_{c}$	${\mathrm{W}}_{a}$	${\mathrm{W}}_{b}$	${\mathrm{W}}_{c}$
$\mathrm{L}.\mathrm{lnEEE}$	0.7690*** (40.53)	0.7740*** (46.03)	0.7608*** (43.60)	0.7741*** (41.95)	0.7734*** (46.25)	0.7689*** (43.51)
$\mathrm{L}.\mathrm{WlnEEE}$	− 0.0787 (− 1.31)	0.3383* (1.81)	0.0941 (1.16)	− 0.0761 (− 1.28)	0.2852 (1.52)	0.1110 (1.32)
$\mathrm{lnGTI}$	0.0077 (1.35)	0.0048 (0.93)	0.0027 (0.50)	0.0084 (1.48)	0.0021 (0.39)	− 0.0020 (− 0.36)
$\mathrm{lnGTI}2$	0.0017*** (3.90)	0.0014*** (3.50)	0.0013*** (2.78)	0.0016*** (3.60)	0.0010*** (2.60)	0.0008 (1.60)
$\mathrm{W}$*$\mathrm{lnGTI}$	− 0.0162*** (− 3.07)	− 0.1272*** (− 4.99)	− 0.0135 (− 1.16)	− 0.0223*** (− 4.24)	− 0.1083*** (− 4.28)	− 0.0122 (− 1.05)
$\mathrm{lnER}$				− 0.0105** (− 2.67)	− 0.0075** (− 2.06)	− 0.0055 (− 1.46)
$\mathrm{W}$*$\mathrm{lnER}$				0.0231*** (3.18)	0.0609*** (3.08)	− 0.0086 (− 0.69)
$\mathrm{lnGTI}$*$\mathrm{lnER}$				− 0.0000 (− 0.22)	0.0001 (0.63)	0.0003* (1.80)
$\mathrm{W}$*$\mathrm{lnGTI}$*$\mathrm{lnER}$				0.0014*** (6.09)	0.0031*** (3.13)	0.0006 (1.19)
$\mathrm{lnControls}$	Yes	Yes	Yes	Yes	Yes	Yes
Province/Year	Yes	Yes	Yes	Yes	Yes	Yes
Spatial rho	0.0482 (0.69)	0.4532** (2.33)	0.1268 (1.50)	0.0080 (0.11)	0.4498** (2.29)	0.1548* (1.78)
Sigma2_e	0.0004*** (15.50)	0.0003*** (15.74)	0.0004*** (15.62)	0.0003*** (15.51)	0.0003*** (15.73)	0.0004*** (15.64)
R-squared	0.980	0.854	0.971	0.974	0.933	0.961
Observations	420	420	420	420	420	420

z-statistics in parentheses, ***, **, * indicate significance at the level of 1%, 5%, 10%.

**Table 4:** Results of direct, indirect, and total effects of GTI on EEE under ER in the short-term.

Weight	${\mathrm{W}}_{a}$			${\mathrm{W}}_{b}$			${\mathrm{W}}_{c}$		
Effect	Direct	Indirect	Total	Direct	Indirect	Total	Direct	Indirect	Total
$\mathrm{lnGTI}$	0.0089 (1.60)	− 0.0219*** (− 4.11)	− 0.0130** (− 2.02)	0.0045 (0.88)	− 0.0786*** (− 3.83)	− 0.0741*** (− 3.62)	− 0.0013 (− 0.24)	− 0.0106 (− 0.99)	− 0.0119 (− 1.03)
$\mathrm{lnGTI}2$	0.0016*** (3.58)	0.0000 (0.19)	0.0016*** (3.52)	0.0010*** (2.60)	− 0.0003* (− 1.82)	0.0007** (2.39)	0.0008 (1.61)	− 0.0001 (− 1.10)	0.0007 (1.61)
$\mathrm{lnER}$	− 0.0104*** (− 2.75)	0.0230*** (3.11)	0.0126* (1.84)	− 0.0087** (− 2.41)	0.0479*** (2.92)	0.0392** (2.49)	− 0.0053 (− 1.47)	− 0.0057 (− 0.52)	− 0.0110 (− 0.93)
$\mathrm{lnGTI}$*$\mathrm{lnER}$	− 0.0000 (− 0.17)	0.0014*** (5.78)	0.0014*** (5.23)	0.0000 (0.28)	0.0021*** (2.76)	0.0022*** (2.87)	0.0003* (1.81)	0.0005 (1.07)	0.0008 (1.49)
$\mathrm{lnControls}$	Yes			Yes			Yes		
Province/Year	Yes			Yes			Yes		
Spatial rho	0.0080 (0.11)			0.4498** (2.29)			0.1548* (1.78)		
sigma2_e	0.0003*** (15.51)			0.0003*** (15.73)			0.0004*** (15.64)		
R-squared	0.974			0.933			0.961		
Observations	420			420			420		

z-statistics in parentheses, ***, **, * indicate significance at the level of 1%, 5%, 10%.

**Table 5:** Results of direct, indirect, and total effects of SubGI, SymGI on EEE under ER in the short-term.

Weight	${\mathrm{W}}_{a}$			${\mathrm{W}}_{b}$			${\mathrm{W}}_{c}$		
Effect	Direct	Indirect	Total	Direct	Indirect	Total	Direct	Indirect	Total
$\mathrm{lnSubGI}$	0.0128** (2.44)	− 0.0304*** (− 2.78)	− 0.0176 (− 1.43)	0.0106** (2.09)	− 0.1151*** (− 3.91)	− 0.1045*** (− 3.44)	0.0143*** (2.69)	− 0.0270** (− 2.19)	− 0.0127 (− 0.94)
$\mathrm{lnSymGI}$	− 0.0200*** (− 3.50)	0.0179 (1.56)	− 0.0021 (− 0.17)	− 0.0191*** (− 3.42)	0.0578* (1.90)	0.0387 (1.29)	− 0.0266*** (− 4.43)	0.0216 (1.50)	− 0.0050 (− 0.34)
$\mathrm{lnER}$	− 0.0087** (− 2.20)	0.0266*** (3.49)	0.0179** (2.54)	− 0.0067* (− 1.80)	0.0519*** (3.11)	0.0452*** (2.85)	− 0.0017 (− 0.44)	− 0.0084 (− 0.70)	− 0.0101 (− 0.78)
$\mathrm{lnSubGI}$*$\mathrm{lnER}$	− 0.0011 (− 1.46)	0.0030* (1.88)	0.0019 (1.07)	− 0.0007 (− 0.96)	0.0087** (2.01)	0.0080* (1.83)	− 0.0013* (− 1.70)	− 0.0003 (− 0.10)	− 0.0016 (− 0.62)
$\mathrm{lnSymGI}$*$\mathrm{lnER}$	0.0013 (1.56)	− 0.0020 (− 1.19)	− 0.0007 (− 0.39)	0.0009 (1.07)	− 0.0072 (− 1.53)	− 0.0063 (− 1.34)	0.0018** (2.06)	0.0009 (0.33)	0.0027 (0.94)
$\mathrm{lnContrils}$	Yes			Yes			Yes		
Province/Year	Yes			Yes			Yes		
Spatial rho	0.0248 (0.34)			0.4067** (2.09)			0.0863 (1.02)		
sigma2_e	0.0003*** (15.51)			0.0003*** (15.70)			0.0004*** (15.63)		
R-squared	0.972			0.952			0.964		
Observations	420			420			420		

z-statistics in parentheses, ***, **, * indicate significance at the level of 1%, 5%, 10%.

Table 4 presents the results of the direct, spatial, and total effects of GTI on EEE considering the impact of ER. The coefficients of the total effect of GTI are significantly negative under the weighting matrices ${W}_{a}$ and ${W}_{b}$ ^65^. Furthermore, the total effect follows the same trend as the spatial effect, indicating that the inhibitory effect of GTI on neighboring regions is greater than its promoting effect on the local region^66^. One possible reason for this is that the promotion of GTI leads to resource competition in neighboring areas. What sets this study apart from others is the consideration of the quadratic term of GTI^26,47^. The study finds that the coefficient for the quadratic term of GTI is significantly positive^30^ and consistent with the trend of direct effects. This suggests that there is a critical point in the impact of GTI on EEE. Before reaching this critical point, the negative spatial spillover effect of GTI on EEE is greater than the positive direct effect, leading to a decrease in EEE. However, after reaching the critical point, the positive direct effect of GTI on EEE outweighs the negative spatial spillover effect, thereby improving EEE. The main reason for this is that as GTI progresses, its required conditions become more refined, and good cooperative relationships develop among neighboring regions, accelerating the development of green technology in various regions.

**Figure 3:**
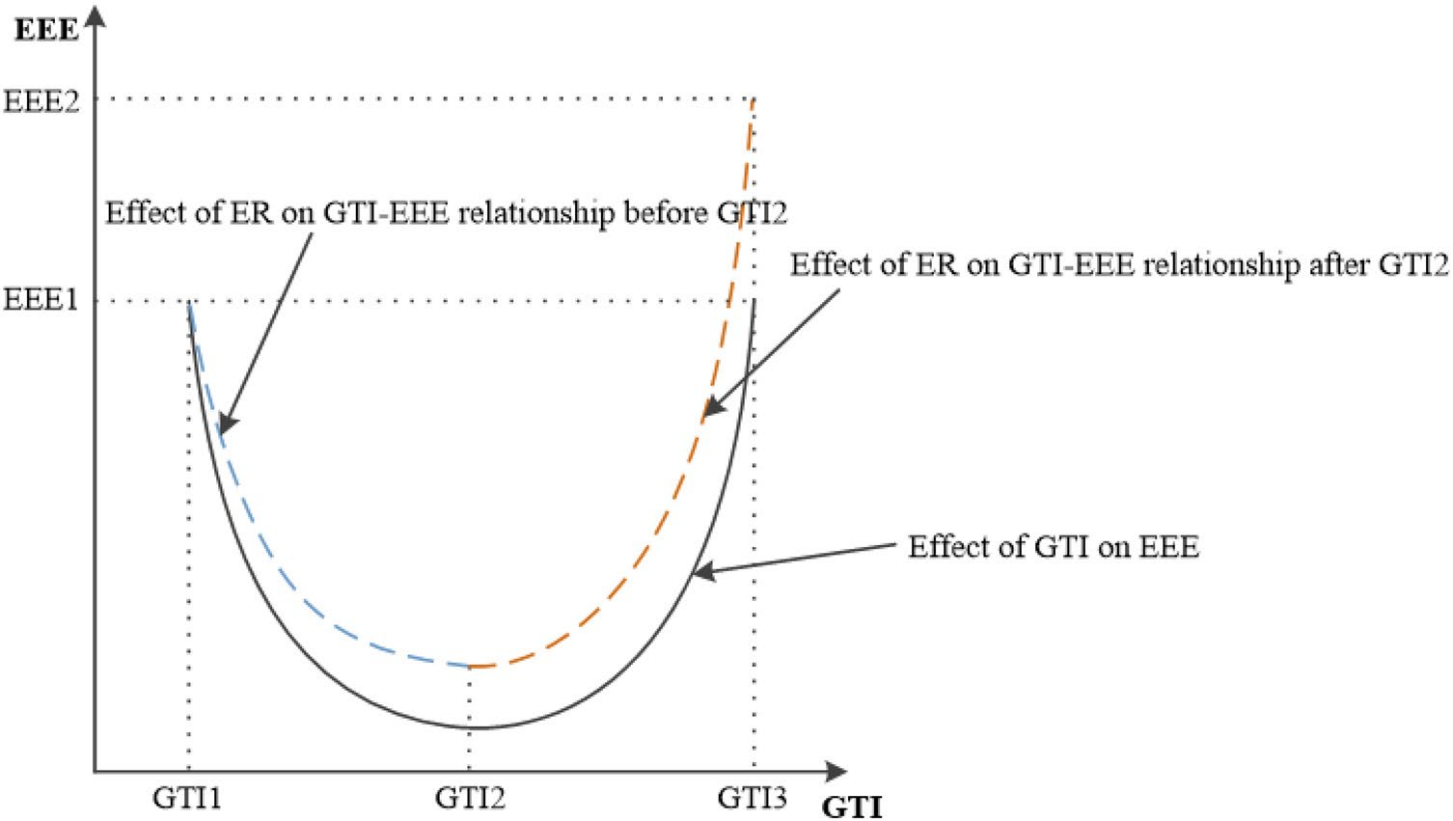
Moderating effect of ER on the relationship between GTI and EEE.

Similarly, under matrices ${W}_{a}$ and ${W}_{b}$, the total effect coefficient of ER on EEE is significantly positive, indicating that the implementation of ER can enhance EEE^19^.One possible reason for this result is that this study uses the ratio of emission fees to GDP as the measurement indicator for ER. Excessive emissions by enterprises lead to higher emission costs, increased operating costs for businesses, thereby prompting companies to update production equipment^35^, reduce pollutant emissions, and result in a decrease in EEE. Furthermore, the coefficient for the interaction term between ER and GTI is significantly positive, but the impact of GTI on EEE follows a U-shaped curve. This suggests that before GTI reaches its critical point, the implementation of ER mitigates the negative impact of GTI on EEE. The reason for this could be that in the early stages of GTI implementation, businesses face high sunk costs, and the implementation of environmental policies encourages businesses and individuals to invest in the research and application of green technology, thereby increasing financial support for green technology development. After GTI reaches its critical point, the implementation of ER strengthens the promoting effect of GTI on EEE. The primary reason is that the conditions required for green technology innovation are already mature, and businesses have developed awareness of green technology innovation. At this point, the implementation of ER can stimulate long-term investment and collaboration by businesses, accelerating the development and application of green technology. As illustrated in Fig. 3.

While we have gained further insights into the impact of GTI on EEE and the role of ER in this effect, the specific source of GTI’s influence on EEE remains unknown. This is because GTI encompasses both the development of new energy technologies and improvement of environmental protection and resource recycling technologies. To unravel this mystery, the study decomposed GTI into SubGI and SymGI and conducted a new empirical analysis, as shown in Table 5. Under the weight matrix ${W}_{b}$, the coefficient of SubGI is significantly negative in the total effect, consistent with the trend observed in the GTI coefficient in Table 4. However, the coefficient of SymGI is not significant. This indicates that the effect of GTI on EEE is primarily driven by SubGI^31^. Which means GTI affects EEE mainly through the development and expansion of new clean energy technologies^11^. The possible reason is that new clean energy technologies not only reduce resource wastage but also reduce dependence on traditional fossil fuels, further reducing greenhouse gas emissions^66^. Furthermore, the role of ER and its effect on SubGI’s impact on EEE remain consistent with the results before the decomposition of GTI.

### Estimated results after EEE decomposition

To further estimate which component of EEE is influenced by GTI, as EEE is composed of PTE and SE, this study decomposed EEE into PTE and SE and conducted new empirical analysis, as shown in Table 6. Under the three spatial weight matrices, the coefficients of the explanatory variables corresponding to SE are almost non-significant, indicating that the impact of GTI on EEE is primarily manifested in PTE. Therefore, this research takes PTE as the dependent variable and decomposes GTI into SubGI and SymGI for re-estimation, with results shown in Table 7.

**Table 6:** Results of the impact of GTI on PTE and SE under ER.

Model	(8)			(10)		
Weight	${\mathrm{W}}_{a}$	${\mathrm{W}}_{b}$	${\mathrm{W}}_{c}$	${\mathrm{W}}_{a}$	${\mathrm{W}}_{b}$	${\mathrm{W}}_{c}$
$\mathrm{L}.\mathrm{lnPTE}$	0.7760*** (35.32)	0.7699*** (38.09)	0.7456*** (35.50)			
$\mathrm{L}.\mathrm{WlnPTE}$	− 0.1044 (− 1.54)	0.2874 (1.37)	0.1553** (2.08)			
$\mathrm{L}.\mathrm{lnSE}$				0.7770*** (20.20)	0.7737*** (18.84)	0.7737*** (18.84)
$\mathrm{L}.\mathrm{WlnSE}$				− 0.1924* (− 1.80)	0.2212 (0.63)	0.2212 (0.63)
$\mathrm{lnGTI}$	− 0.0005 (− 0.06)	− 0.0010 (− 0.14)	0.0015 (0.19)	0.0019 (0.35)	− 0.0017 (− 0.32)	− 0.0017 (− 0.32)
$\mathrm{lnGTI}2$	0.0006 (1.03)	0.0005 (0.85)	0.0011 (1.51)	0.0001 (0.23)	0.0000 (0.10)	0.0000 (0.10)
$\mathrm{W}$*$\mathrm{lnGTI}$	− 0.0194*** (− 2.71)	− 0.0990*** (− 2.88)	0.0202 (1.30)	− 0.0065 (− 1.17)	− 0.0193 (− 0.74)	− 0.0193 (− 0.74)
$\mathrm{lnER}$	− 0.0070 (− 1.32)	− 0.0026 (− 0.53)	0.0010 (0.19)	− 0.0057 (− 1.44)	− 0.0062* (− 1.70)	− 0.0062* (− 1.70)
$\mathrm{W}$*$\mathrm{lnER}$	0.0208** (2.26)	0.0654** (2.55)	0.0083 (0.53)	− 0.0017 (− 0.26)	− 0.0069 (− 0.35)	− 0.0069 (− 0.35)
$\mathrm{lnGTI}$*$\mathrm{lnER}$	0.0005* (1.94)	0.0007*** (2.74)	0.0005** (2.14)	− 0.0003 (− 1.41)	− 0.0002 (− 1.24)	− 0.0002 (− 1.24)
$\mathrm{W}$*$\mathrm{lnGTI}$*$\mathrm{lnER}$	0.0021*** (4.54)	0.0025* (1.77)	0.0006 (0.82)	− 0.0002 (− 0.45)	0.0007 (0.71)	0.0007 (0.71)
$\mathrm{lnControls}$	Yes	Yes	Yes	Yes	Yes	Yes
Province/Year	Yes	Yes	Yes	Yes	Yes	Yes
Spatial rho	0.0352 (0.45)	0.5703*** (2.66)	0.2225*** (2.91)	0.0618 (0.74)	0.7113*** (3.05)	0.7113*** (3.05)
sigma2_e	0.0006*** (15.46)	0.0006*** (15.60)	0.0006*** (15.52)	0.0003*** (15.52)	0.0003*** (15.26)	0.0003*** (15.26)
R-squared	0.954	0.924	0.917	0.957	0.968	0.968
Observations	420	420	420	420	420	420

z-statistics in parentheses, ***, **, * indicate significance at the level of 1%, 5%, 10%.

**Table 7:** Results of direct, indirect, and total effects of SubGI, SymGI on PTE under ER in the short-term.

Weight	${\mathrm{W}}_{a}$			${\mathrm{W}}_{b}$			${\mathrm{W}}_{c}$		
Effect	Direct	Indirect	Total	Direct	Indirect	Total	Direct	Indirect	Total
$\mathrm{lnSubGI}$	0.0102* (1.66)	− 0.0211* (− 1.79)	− 0.0109 (− 0.83)	0.0121** (2.02)	− 0.0812*** (− 2.64)	− 0.0691** (− 2.21)	0.0126** (2.01)	− 0.0237* (− 1.67)	− 0.0111 (− 0.72)
$\mathrm{lnSymGI}$	− 0.0219*** (− 3.19)	0.0079 (0.60)	− 0.0140 (− 0.99)	− 0.0200*** (− 2.86)	0.0233 (0.71)	0.0033 (0.10)	− 0.0247*** (− 3.52)	0.0526*** (3.03)	0.0280 (1.60)
$\mathrm{lnER}$	− 0.0069 (− 1.21)	0.0253*** (2.67)	0.0184** (2.14)	− 0.0035 (− 0.65)	0.0516** (2.56)	0.0481*** (2.59)	0.0040 (0.77)	0.0035 (0.24)	0.0075 (0.49)
$\mathrm{lnSubGI}$*$\mathrm{lnER}$	− 0.0009* (− 1.93)	0.0014 (1.36)	0.0005 (0.41)	− 0.0009* (− 1.87)	0.0053* (1.90)	0.0044 (1.48)	− 0.0007 (− 1.53)	0.0034 (1.49)	0.0027 (1.14)
$\mathrm{lnSymGI}$*$\mathrm{lnER}$	0.0014*** (2.73)	0.0006 (0.58)	0.0020 (1.64)	0.0015*** (2.91)	− 0.0035 (− 1.31)	− 0.0020 (− 0.73)	0.0013*** (2.63)	− 0.0028 (− 1.14)	− 0.0015 (− 0.60)
$\mathrm{lnControls}$	Yes			Yes			Yes		
Province/Year	Yes			Yes			Yes		
Spatial rho	0.0199 (0.26)			0.5156** (2.42)			0.1762** (2.36)		
sigma2_e	0.0006*** (15.48)			0.0005*** (15.60)			0.0006*** (15.52)		
R-squared	0.959			0.923			0.901		
Observations	420			420			420		

z-statistics in parentheses, ***, **, * indicate significance at the level of 1%, 5%, 10%.

Under the spatial weight matrix ${W}_{b}$, the total effect of SubGI on PTE is significantly negative, consistent with the direction of the spatial effect, indicating that SubGI’s impact on the PTE of neighboring areas is greater than on the local area. Additionally, the total effect of SymGI on PTE is not significant, suggesting that GTI’s impact on PTE is also driven by SymGI^47,49^. The possible reason is that the development of SubGI requires a significant capital and labor input, and as neighboring areas, some of the capital and labor within the province will also be partially absorbed, leading to a decrease in PTE given the allocated resources. In contrast, under the interaction of direct and indirect effects, ER did not play a role in the impact of SymGI on PTE. The possible reason for this is that the implementation of ER increases the emission costs for local businesses, reduces the incentive for local businesses to develop GTI, and subsequently slows down the competition for capital and labor in neighboring areas, leading to a mutual offset between the two effects.

### The robustness test

To ensure the reliability and accuracy of the methodology adopted and the conclusions obtained in this article, a robustness test was conducted by replacing the control variables and the explanatory variable with a one-period lag.

Firstly, in the robustness test involving the replacement of control variables, fiscal technology expenditure (FST) was substituted for R&D investment intensity. The effects of GTI, SubGI, SymGI, ER and the interaction term of ER with GTI, SubGI and SymGI on EEE as well as PTE are re-evaluated in this study under short-term effects based on the DSDM and the geographical distance weight matrix (${\mathrm{W}}_{b}$), respectively. The results presented in Table 8 show that all outcomes after the replacement of control variables maintain the same positive or negative signs and levels of significance as the original results. The coefficients exhibit only minor changes when comparing before and after the substitution. This suggests a high degree of robustness in the findings, thereby confirming the reliability of our estimates.

**Table 8:** Results of robustness tests for replacement variables.

Model	(6)			(7)			(8)			(9)		
Effect	Direct	Indirect	Total	Direct	Indirect	Total	Direct	Indirect	Total	Direct	Indirect	Total
$\mathrm{lnGTI}$	0.0065 (1.25)	− 0.0753*** (− 3.75)	− 0.0687*** (− 3.41)				0.0023 (0.32)	− 0.0659*** (− 2.64)	− 0.0637** (− 2.56)			
$\mathrm{lnGTI}2$	0.0009** (2.36)	− 0.0003* (− 1.74)	0.0007** (2.19)				0.0005 (0.85)	− 0.0002 (− 0.79)	0.0003 (0.84)			
$\mathrm{lnSubGI}$				0.0107** (2.12)	− 0.1175*** (− 3.98)	− 0.1068*** (− 3.51)				0.0121** (2.01)	− 0.0819*** (− 2.66)	− 0.0698** (− 2.23)
$\mathrm{lnSymGI}$				− 0.0166*** (− 2.98)	0.0641** (2.06)	0.0475 (1.53)				− 0.0197*** (− 2.83)	0.0217 (0.66)	0.0019 (0.06)
$\mathrm{lnER}$	− 0.0101*** (− 2.79)	0.0492*** (3.03)	0.0391** (2.51)	− 0.0084** (− 2.26)	0.0539*** (3.24)	0.0455*** (2.88)	− 0.0045 (− 0.90)	0.0471** (2.45)	0.0426** (2.36)	− 0.0038 (− 0.69)	0.0520*** (2.58)	0.0482*** (2.59)
$\mathrm{lnGTI}$*$\mathrm{lnER}$	− 0.0000 (− 0.11)	0.0028*** (3.28)	0.0028*** (3.36)				0.0006** (2.52)	0.0016 (1.47)	0.0022** (2.07)			
$\mathrm{lnSubGI}$*$\mathrm{lnER}$				− 0.0006 (− 0.80)	0.0091** (2.09)	0.0085* (1.94)				− 0.0009* (− 1.88)	0.0052* (1.88)	0.0042 (1.46)
$\mathrm{lnSymGI}$*$\mathrm{lnER}$				0.0007 (0.85)	− 0.0070 (− 1.51)	− 0.0063 (− 1.35)				0.0015*** (2.91)	− 0.0034 (− 1.27)	− 0.0019 (− 0.68)
$\mathrm{lnControls}$	Yes			Yes			Yes			Yes		
Province/Year	Yes			Yes			Yes			Yes		
Spatial rho	0.4592** (2.33)			0.4166** (2.13)			^0^^.5709^*** ^(2.66)^			0.5157** (2.42)		
sigma2_e	0.0003*** (15.73)			0.0003*** (15.71)			^0.0006^*** ^(15.60)^			0.0005*** (15.60)		
R-squared	0.943			0.958			0.924			0.918		

z-statistics in parentheses, ***, **, * indicate significance at the level of 1%, 5%, 10%.

Secondly, in the short-term effects, the explanatory variables’ spatial Durbin lag one-period models were employed, along with the geographical distance weight matrix (${\mathrm{W}}_{b}$), to reassess the impact of GTI, SubGI, SymGI, ER, as well as the interactions between ER and GTI, SubGI, and SymGI on EEE and PTE. Similarly, the results in Table 9 demonstrate that all outcomes using lagged one-period explanatory variables maintain the same positive or negative signs and levels of significance as the dynamic Durbin model’s results. Coefficients exhibit only minor changes, reaffirming the strong robustness of the findings and the reliability of our estimates.

**Table 9:** Results of lagged one-period robustness tests for the explanatory variables.

Model	(6)			(7)			(8)			(9)		
Effect	Direct	Indirect	Total	Direct	Indirect	Total	Direct	Indirect	Total	Direct	Indirect	Total
$\mathrm{lnGTI}$	0.0647*** (5.47)	0.1073 (1.50)	0.1720** (2.38)				0.0002 (0.03)	− 0.0579** (− 2.29)	− 0.0577** (− 2.30)			
$\mathrm{lnGTI}2$	0.0032*** (3.64)	− 0.0003 (− 0.38)	0.0030*** (2.78)				0.0005 (0.88)	− 0.0001 (− 0.83)	0.0003 (0.88)			
$\mathrm{lnSubGI}$				0.0092* (1.78)	− 0.1280*** (− 4.00)	− 0.1187*** (− 3.58)				0.0109* (1.80)	− 0.0796** (− 2.43)	− 0.0686** (− 2.05)
$\mathrm{lnSymGI}$				− 0.0178*** (− 3.04)	0.0742** (2.24)	0.0564* (1.71)				− 0.0194*** (− 2.72)	0.0321 (0.91)	0.0127 (0.36)
$\mathrm{lnER}$	− 0.0133 (− 1.59)	0.1045** (2.16)	0.0912* (1.91)	− 0.0071* (− 1.93)	0.0589*** (3.27)	0.0518*** (2.97)	− 0.0049 (− 0.99)	0.0499** (2.57)	0.0450** (2.46)	− 0.0042 (− 0.78)	0.0564*** (2.66)	0.0522*** (2.63)
$\mathrm{lnGTI}$*$\mathrm{lnER}$	− 0.0012*** (− 3.68)	0.0054** (2.22)	0.0042* (1.74)				0.0006*** (2.76)	0.0019* (1.82)	0.0025** (2.42)			
$\mathrm{lnSubGI}$*$\mathrm{lnER}$				− 0.0006 (− 0.80)	0.0101** (2.12)	0.0095** (1.97)				− 0.0009* (− 1.75)	0.0047 (1.61)	0.0038 (1.22)
$\mathrm{lnSymGI}$*$\mathrm{lnER}$				0.0008 (0.95)	− 0.0083 (− 1.60)	− 0.0075 (− 1.44)				0.0015*** (2.95)	− 0.0026 (− 0.94)	− 0.0011 (− 0.38)
$\mathrm{lnControls}$	Yes			Yes			Yes			Yes		
Province/Year	Yes			Yes			Yes			Yes		
Spatial rho	0.1323 (0.60)			0.2108** (2.16)			0.4364*** (3.44)			0.3690*** (2.85)		
sigma2_e	0.0019*** (15.52)			0.0003*** (15.46)			0.0006*** (15.65)			0.0006*** (15.67)		
R-squared	0.265			0.967			0.952			0.953		

z-statistics in parentheses, ***, **, * indicate significance at the level of 1%, 5%, 10%.

## Conclusion and policy implications

### Conclusion

This study utilized data spanning from 2003 to 2017 from 30 Chinese provinces and employed the NDDF method to calculate EEE for each province. Furthermore, considering the inherent nature of GTI, we decomposed it into two sub-indices, SubGI and SymGI. Through the use of DSDM, we investigated the impact of GTI on EEE, the sources of this impact, and the role of ER in influencing this relationship. The key findings are as follows:

1. The impact of GTI on EEE follows a U-shaped pattern, initially leading to a decrease in EEE, followed by an increase. Furthermore, this effect is primarily attributed to the influence of SubGI on EEE.
2. SubGI reduces EEE, with this effect being more pronounced in neighboring regions. Specifically, the promotion of EEE by SubGI within the local area is less significant than its inhibitory effect on EEE in neighboring regions.
3. While ER can enhance EEE, due to the U-shaped impact of GTI on EEE, it leads to ER weakening the negative effect of GTI on EEE before reaching the critical point. However, after reaching the critical point, ER strengthens the promotion effect of GTI on EEE.
4. The impact of GTI on EEE is primarily manifested in the PTE component, and this effect is primarily driven by SubGI.

### Policy implications

Currently, China is in a period of rapid economic development, and this process is characterized by a high level of energy dependence. This phenomenon has placed significant pressure on achieving “peak carbon emissions” and “carbon neutrality”. To ensure that the development of green technology innovation and environmental policies can better improve energy-environmental efficiency in China and other developing countries, this study proposes the following policy implications:

1. Enhancing green technology innovation should prioritize the development and utilization of clean energy. Establishing a comprehensive incentive mechanism for green technology innovation is essential to encourage the transformation and upgrading of existing technologies, leading to reduced energy consumption and decreased carbon emissions.
2. Prudent control over the intensity of environmental regulations is essential. Tailoring environmental regulation policies to the development status of different regions, such as command-based environmental regulatory policies, market-based environmental regulatory policies, and voluntary-based environmental regulatory policies.
3. Governments at all levels should prioritize regional collaborative governance with the aim of enhancing energy-environmental efficiency. Seeking breakthroughs in addressing environmental and greenhouse gas emission governance issues.

However, this study has some limitations that should be addressed in future research to enrich this field. Firstly, this study used provincial-level data as research samples, making the conclusions relatively macroscopic. Therefore, future evaluations of GTI and ER should aim to be conducted at the city or enterprise level to delve deeper into the practical realities of GTI and ER. Secondly, due to limitations in statistical data, the study data covers the period from 2003 to 2017, lacking insights into recent years. Future work can improve this by using alternative indicators or changing calculation methods. Additionally, this study measured ER using pollution fees. In future research, different measures can be explored to classify ER into market-based ER, command-and-control ER, and more, to investigate the varied roles of different types of ER.

## Data Availability

The datasets generated during and/or analyzed during the current study are available from the corresponding author on reasonable request.

## Appendix

See Table 10.

**Table 10:** Variable type and definitions.

Type	Variable	Symbol	Definition
Explained variables	Energy-environmental efficiency	EEE	Measured by non-radial directional distance function
	Scale efficiency	SE	Measured by non-radial directional distance function
	Pure technical efficiency	PTE	Measured by non-radial directional distance function
Explanatory variables	Green technology innovation	GTI	The sum of the number of green invention patent applications and the number of green utility patent applications
	Substantive green innovation	SubGI	Number of green invention patent applications
	Symbolic green innovation	SymGI	Number of green utility patent applications
	environmental regulation	ER	The proportion of the total emission fee to GDP
Control variables	Gross domestic product per capita	PGDP	The ratio of GDP to total population
	Industrial structure	IS	The value added to GDP ratio of secondary industry
	Foreign direct investment	FDI	The ratio of foreign direct investment to GDP
	Energy consumption structure	ECStruc	The ratio of coal usage to total energy consumption
	Urbanization level	Urban	The ratio of urban living population to total population
	R&D investment intensity	RDI	R&D expenditure
	Energy intensity	EI	Energy consumption per unit of GDP
	Fixed asset investment	Fix	Total investment in fixed assets
	R&D personnel input	RDP	R&D full time equivalent
